# ﻿*Corynoneura* Winnertz species from Hunan Province, Oriental China, delineated with morphological and 16S rDNA data (Diptera, Chironomidae)

**DOI:** 10.3897/zookeys.1082.73019

**Published:** 2022-01-19

**Authors:** Yue Fu, Xiang-Liang Fang, Xin-Hua Wang, Mi Shen, Yun-Li Xiao

**Affiliations:** 1 Hubei Key Laboratory of Economic Forest Germplasm Improvement and Resources Comprehensive Utilization, Hubei Collaborative Innovation Center for the Characteristic Resources Exploitation of Dabie Mountains, Hubei Zhongke Research Institute of Industrial Technology, Huanggang Normal University, Huanggang City, Hubei, 438000, China Huanggang Normal University Huanggang China; 2 College of Life Sciences, Nankai University, Tianjin 300071, China Nankai University Tianjin China

**Keywords:** Mitochondrial gene, morphology, non-biting midge, taxonomy

## Abstract

The genus *Corynoneura* Winnertz, 1846 from Hunan Province in Oriental China is reviewed. Four new species, *C.enormis* Fu **sp. nov.**, *C.gibbera* Fu **sp. nov.**, *C.incuria* Fu **sp. nov.**, and *C.longshanensis* Fu **sp. nov.** are described and illustrated based on adult males. Sequence data from the 16S rDNA gene were used to infer relationships between these species and complement morphological delineation. Sequences from the mitochondrial large ribosomal subunit (16S rDNA) from these species are uploaded to the National Center for Biotechnology Information (NCBI). Relationships were inferred using the Neighbor-Joining method based on 16S rDNA.

## ﻿Introduction

*Corynoneura* was erected by Winnertz (1846) with *Corynoneurascutellata* Winnertz, 1846 as the type species. Fu et al. (2009) and Fu and Sæther (2012) reviewed the East Asia and Nearctic members of this genus. In addition, the different life stages of species of the genus from different geographical areas were studied by a number of authors (Schlee 1968; Wiedenbrug and Trivinho-Strixino 2011; Wiedenbrug et al. 2012; Moubayed-Breil 2015; Makarchenko et al. 2019).

Prior to this study, there were 107 valid species in the world, including 47 species from the Palearctic Region, 19 species from the Nearctic Region, 25 species from the Neotropical Region, 27 species from the Oriental Region, four species from the Afrotropical Region, and five species from the Australasian Region (Ashe and O’Connor 2012; Fu and Sæther 2012; Fu et al. 2018, 2019, 2020; Makarchenko et al. 2019).

Previously, only one species, namely *Corynoneuraprominens* Fu, Sæther & Wang, 2009 was recorded in Hunan Province. In this study, four new species are described and illustrated based on the new material from Hunan Province: *Corynoneuraenormis* sp. nov., *Corynoneuragibbera* sp. nov., *Corynoneuraincuria* sp. nov. and *Corynoneuralongshanensis* sp. nov.. The female of *C.incuria* sp. nov., associated with the male by 16S rDNA, is described and illustrated.

## ﻿Materials and methods

Adults were mainly collected in the habitats of small streams and lakes next to mountain forests. Adults were collected by light traps near the water body or swept from marginal vegetation beside natal aquatic sites. The specimens were preserved in 85% ethanol, and stored in the dark at 4 °C before molecular analyses. Total genomic DNA of specimens was extracted from the thorax and legs using Qiagen DNA Blood & Tissue Kit. The standard protocol of the Qiagen DNeasy Blood & Tissue Kit was used, except that the final elution volume was 100 𝜇L due to the small specimen size. PCR amplification of the mitochondrial 16S ribosomal RNA gene was carried out with the primers and temperature regimes given in Ekrem et al. (2010). After DNA extraction, the clear exoskeleton was washed with 96% ethanol and mounted in Euparal on microscope slides together with the corresponding antennae, head, wings, and legs following the procedure outlined by Sæther (1969). Morphological nomenclature follows Sæther (1980).

Measurements are given as ranges followed by the mean, when three or more specimens were measured. The specimens examined in this study are deposited at the College of Biology and Agricultural Resources, Huanggang Normal University (**HNU**), Huanggang, China.

### ﻿Abbreviations used in text as follows:

**AR** antennal ratio = length of ultimate flagellomere/combined lengths of flagellomeres one to penultimate;

**VR** venarum ratio;

**Cu** cubitus;

**P_1_, P_2_, P_3_** fore, middle, and hind legs, respectively;

**fe** femur;

**ti** tibia;

**ta** tarsomere;

**LR** leg ratio (ratio of metatarsus to tibia in front leg);

**BV** Bein ratio (length of (femur + tibia + ta_1_) /length of (ta_2_ + ta_3_ + ta_4_ + ta_5_));

**SV** Schenkel-Schiene ratio (length of (femur + tibia) / length of ta);

**BR** bristle ratio (ratio of longest seta on ta1 to minimum width of ta_1_ measured one third from apex);

**HR** hypopygium ratio = gonocoxite length / gonostylus length;

**HV** hypopygium value =body length / gonostylus length × 10.

Measurements and ratios of hind tibia follow Schlee (1968) as follows:

**a** Maximum width;

**b** Length of ventral elongation;

**c_1_** Length of strong broad part, measured from apex;

**c_2_** Total length of broadening;

**d** Width of tibia basally to the apical broadening.

## ﻿Results

### ﻿Taxonomic account

#### ﻿ Corynoneura

Taxon classificationAnimaliaDipteraChironomidae

Winnertz, 1846

89423E28-B548-5C10-B371-B03A05BBFE50

Corynoneura Winnertz, 1846: 12.

#### ﻿ Corynoneura enormis

Taxon classificationAnimaliaDipteraChironomidae

Fu sp. nov.

DF104396-9D4F-5A50-929F-DBC29A6FF7A2

http://zoobank.org/6D4BD075-EC3F-4BC2-9A53-D86F97A7202D

Figure 1

##### Type material.

***Holotype***, male (HNU: 17091206HJL), China: Hunan Province, Loudi City, Xinhua County, Xihe Town, Cushi Village, 27°51'45"N, 111°31'51"E, 315 m a. s. l., 29.Ⅶ.2016, sweep net, leg. Jingli Huang.

##### Etymology.

From Latin, *enormis*, immense, huge, vast, referring to lateral sternapodeme with a large attachment point.

##### Diagnosis.

The male imago is characterized by having an antenna with eight flagellomeres, AR 0.51; anterior margin of cibarial pump distinctly concave; hind tibia with hooked spur; superior volsella small rounded and undeveloped; inferior volsella narrow, with dented edge, along the inner margin of gonocoxite; phallapodeme apically curved, placed in lateral position of sternapodeme; sternapodeme curved into a U-shape, and lateral sternapodeme with large caudal attachment point.

##### Description.

**Adult males (*N* = 1).** Total length 0.92 mm. Wing length 0.53 mm. Total length/wing length ratio 1.74. Wing length/profemur length ratio 2.69.

***Coloration.*** Head dark brown; thorax dark brown. Legs yellowish. Abdomen brown.

**Figure 1.**
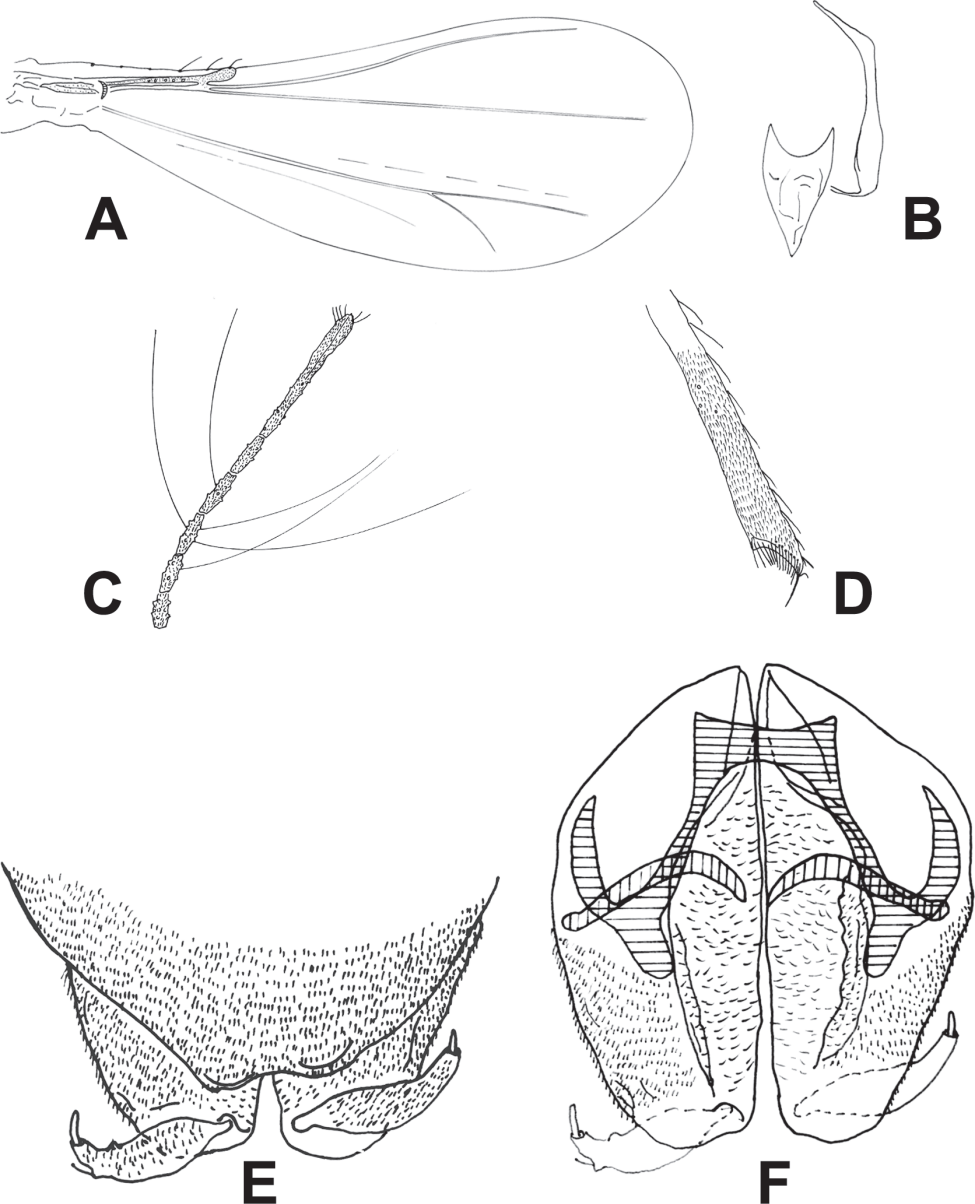
*Corynoneuraenormis* sp. nov., male imago. **A** wing **B** tentorium and cibarial pump **C** apex of antenna **D** legs **E** hypopygium, dorsal view **F** hypopygium, ventral view.

***Head.*** Antenna with eight flagellomeres, AR 0.51, ultimate flagellomere 115 µm long, with many short apical sensilla chaetica (Fig. 1C). Tentorium and cibarial pump as in Figure 1B, tentorium 110 µm long; 12 µm wide; Anterior margin of cibarial pump strongly concave. Clypeus with four setae.

***Thorax.*** Five dorsocentral setae. Scutellum with two setae. One or two prealar setae.

***Wing*** (Fig. 1A). VR 3.0. Cu/wing length ratio 0.54; costa 120 µm long, with five setae; Cu 288 µm long; wing width/wing length ratio 0.45.

***Legs.*** Fore trochanter with dorsal keel. Most of fore- and mid-legs lost. Spurs of hind tibia 25 µm and 10 µm long. Width of hind tibia at apex (a) 29 µm, width of hind tibia ^1^/_3_ from apex (d) 18 µm, elongation length (b) 34 µm, length of maximum thickening (c_1_) 48 µm, total length of thickening (c_2_) 72 µm; hind tibial ratios: a/d 1.61; b/d 1.89; c_1_/d 2.61; c_2_/d 4.00. Hind tibia expanded with comb of 17 setae and S-shaped spur (Fig. 1D). Lengths and other proportions of legs given in Table 1.

**Table 1.** Lengths (in µm) and proportions of leg segments of male *Corynoneuraenormis* sp. nov. (*N* = 1).

	fe	ti	ta_1_	ta_2_	ta_3_	ta_4_	ta_5_	LR	BV	SV	BR
P_1_	197	240	130	71	42	20	28	0.54	3.52	3.36	1.80
P_2_	216	245	145	70	35	16	27	0.59	4.09	3.17	2.00
P_3_	216	211	113	67	29	19	29	0.54	3.75	3.78	1.80

***Hypopygium*** (Fig. 1E, F). Tergite IX medially incurved. Superior volsella small, with rounded margin, anteromedially fused. Inferior volsella along inner margin of gonocoxite with many glandular setae. Phallapodeme scalpel-like, apical curved, 35 µm long, and joint with sternapodeme placed lateral. Transverse sternapodeme 20 µm wide, inverted U-shaped, with small oral projection, lateral sternapodeme with very large attachment point placed and directed caudally. Gonostylus curved tapering, 29 µm long; megaseta 5 µm long. HR 2.31; HV 3.17.

##### Remarks.

This species is similar to *Corynoneuraascensa* Fu & Sæther, 2012 and *Corynoneurasesquipedalis* Fu & Fang, 2018 by having a large attachment point on the lateral sternapodeme. The new species can be separated from *C.ascensa* by having antenna with 8 flagellomeres, narrow and undeveloped inferior volsella; and differs from *C.sesquipedalis* by having a narrow inferior volsella, transverse sternapodeme present and with an oral projection (broad inferior volsella, transverse sternapodeme V-shaped,without transverse part in *C.sesquipedalis*). The sequence of 16S rDNA from this species is highly similar to *Corynoneuratumula* Fu & Fang, 2018, but there are distinct morphological differences between them: in *C.enormis* the antenna has 8 flagellomeres, AR 0.51; inferior volsella narrow, lateral sternapodeme with large caudal attachment point, while *C.tumula* has an antenna with 9 flagellomeres, AR 0.46; inferior volsella relatively broad, lateral sternapodeme with small caudal attachment point.

#### ﻿ Corynoneura gibbera

Taxon classificationAnimaliaDipteraChironomidae

Fu sp. nov.

F11BC3ED-3A98-5343-A989-2CCC6F9BC120

http://zoobank.org/51E7C7EA-3496-4E7D-A6D4-6009D0B8A94E

Figure 2

##### Type material.

***Holotype*** male (HNU: 17090801HJL), China: Hunan Province, Huaihua City, Hecheng County, Wushui River, Xiyi Bridge, 27°33'29"N, 109°57'41"E, 259 m a. s. l., 23.VII.2016, light trap, leg. Haixia Shi. 6 males (HNU: 17090902HJL, 17090904HJL, 17091004HJL, 17091003HJL, 17090804HJL, 17090803HJL), 21–23.VII. 2016, as holotype.

##### Etymology.

From Latin, *gibbera*, protuberant, referring to the prominent inferior volsella.

##### Diagnostic characters.

The male imago is characterized by having an antenna with nine or ten flagellomeres, AR 0.43–0.57, 0.52; superior volsella triangular; inferior volsella prominent, like a small rectangle, and placed caudally of gonocoxite; transverse sternapodeme inverted U-shaped; phallapodeme scalpel-like, in caudal position of sternapodeme.

##### Description.

**Adult male (*N* = 7).** Total length 0.82–1.10, 0.95 mm. Wing length 0.45–0.66, 0.57 mm. Total length/wing length ratio 1.67–1.82, 1.70. Wing length/profemur length ratio 2.32–3.04, 2.79.

***Coloration.*** Head brown, with dark brown eyes; thorax dark brown; legs yellowish; tergites I-V yellowish, VI-IX brownish.

**Figure 2.**
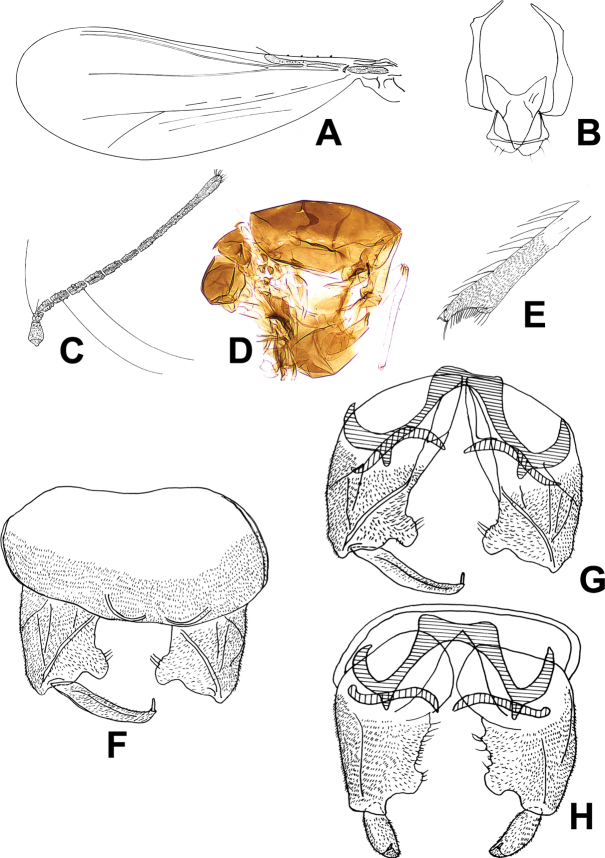
*Corynoneuragibbera* sp. nov., male imago. **A** wing **B** tentorium and cibarial pump **C** antenna **D** thorax **E** hind tibial apex **F** hypopygium, dorsal view **G, H** hypopygium, ventral view.

***Head.*** Antenna with nine or ten flagellomeres, AR 0.43–0.57, 0.52, ultimate flagellomere 98–144, 124 µm long, slightly expanded apically, with many short apical sensilla chaetica (Fig. 2C). Tentorium and cibarial pump as in Figure 2B, tentorium 101–120, 110µm long; 10–26, 15 µm wide; stipes 48–55, 52 µm long, 2–4, 3 µm wide. Anterior margin of cibarial pump strongly concave. Clypeus with 8–10, 9 setae. Length of palpomeres (in µm): 10–12, 11; 12–14, 13; 14–19, 17; 22–26, 24; 32–53, 44. Palpomere 5/3 ratio: 1.71–2.82, 2.14.

***Thorax*** (Fig. 2D). Four or five dorsocentral setae. Scutellum with two setae. One or two prealar setae.

***Wing*** (Fig. 2A). VR 3.0. Cu/wing length ratio 0.44–0.48, 0.46; costa 151–157, 155 µm long, with five or six setae; Cu 255–275, 260 µm long; wing width/wing length ratio 0.38–0.44, 0.42.

***Legs.*** Fore trochanter with dorsal keel. Spurs of fore tibia 17–24, 19 µm long and 7–12, 10 µm long, spurs of mid tibia 7–10, 8 and 10–12, 11 µm long, and spurs of hind tibia 22–29, 24 µm long and 12–14, 13 µm long. Width of fore tibia at apex 17–22, 19 µm, of mid tibia 12–19, 15 µm, of hind tibia (a) 17–24, 22 µm. Width of hind tibia ^1^/_3_ from apex (d) 17–22, 20 µm, elongation length (b) 31–43, 36 µm, length of maximum thickening (c_1_) 60–72, 66 µm, total length of thickening (c_2_) 84–120, 95 µm; hind tibial ratios: a/d 1.41–2.00, 1.60; b/d 1.41–2.39, 2.10; c_1_/d 2.73–4.00, 3.75; c_2_/d 3.83–6.67, 5.62. Hind tibia expanded, with comb of 15–19, 16 setae, with S-shaped spur (Fig. 2E). Lengths and other proportions of legs as in Table 2.

**Table 2.** Lengths (in µm) and proportions of legs segments of male *Corynoneuragibbera* sp. nov. (*N* = 7).

	fe	ti	ta_1_	ta_2_	ta_3_	ta_4_
P_1_	194–225, 208	225–255,245	120–146,131	70–82, 73	38–46,42	19–22, 20
P_2_	265–323,297	245–284, 265	144–176, 162	65–79, 72	34–36, 35	14–19, 17
P_3_	225–265, 243	235–284, 255	120–146, 132	70–77, 75	29–31, 30	17–19, 18
	**ta_5_**	**LR**	**BV**	**SV**	**BR**	
P_1_	26–31, 29	0.51–0.56,0.53	3.35–3.75, 3.55	3.32–3.68, 3.48	1.30–2.20, 1.70	
P_2_	26–31, 29	0.59–0.62, 0.61	4.61–4.88, 4.74	3.40–4.16, 3.64	1.50–2.20, 1.80	
P_3_	26–31, 29	0.51–0.54, 0.52	3.91–4.46, 4.15	3.56–3.92, 3.76	2.00–2.20, 2.10	

***Hypopygium*** (Fig. 2F-H). Tergite IX medially slightly incurved. Superior volsella triangular, with rounded margin. Inferior volsella prominent, rectangular, placed caudally. Phallapodeme scalpel-like, apically curved, 31–36, 34 µm long, in caudal position of sternapodeme. Transverse sternapodeme 17–26, 21 µm wide, with oral projection, inverted U-shaped. Gonostylus relatively long and slender, curved tapering, 26–29, 28 µm long; megaseta 4–5 µm long. HR 2.24–2.48, 2.38; HV 3.15–3.79, 3.39.

##### Remarks.

This species is closely related to *Corynoneuramacula* Fu & Sæther, 2012 by having similarly shaped inferior volsella and an inverted U-shaped sternapodeme. The new species can be separated from the latter by having AR 0.43–0.57, 0.52, gonostylus relatively long and slender, apically curved, while *C.macula* has a yellowish antenna with a dark brown apical spot, AR 0.27–0.37, and the gonostylus is relatively short and strongly curved. The new species is also similar to *Corynoneuraaurora* Makarchenko & Makarchenko, 2010 by having similar inferior volsella, the same shaped sternapodeme and phallapodeme, but differs from the latter by the antenna having 12 flagellomeres, and the gonostylus being slightly convex on the outer edge in *C.aurora*.

#### ﻿ Corynoneura incuria

Taxon classificationAnimaliaDipteraChironomidae

Fu sp. nov.

B4B1ACE9-6F94-5984-9411-51EDA4BAEE72

http://zoobank.org/D06DACE6-EDDD-4172-90AC-E9589D535DAD

Figures 3 , 4

##### Type material.

***Holotype*** male (HNU: 17090903HJL), China: Hunan Province, Huaihua City, Hecheng County, Wushui River, Xiyi Bridge, 29°33'29"N, 109°57'41"E, 259 m a. s. l., 23.VII.2016, light trap, leg. Jingli Huang. ***Paratype***: 1 female (HNU:17091205HJL), CHINA: Hunan Province, Loudi City, Xinhua County, Xihe Town, Cushi Village, 27°51'45"N, 111°31'51"E, 315 m a. s. l., 29.Ⅶ.2016, sweep net, leg. Jingli Huang.

##### Etymology.

From Latin, *incuria*, neglect, referring to the inferior volsella being absent and fused with the inner margin of gonocoxite.

##### Diagnostic characters.

The male imago is characterized by having antenna with eleven flagellomeres, AR 0.31; anterior margin of cibarial pump strongly concave; superior volsella developed and with right-angled corner; inferior volsella almost absent, fused with the inner margin of gonocoxite; transverse sternapodeme curved into U-shaped; phallapodeme scalpel-like, apical slightly curved, placed caudal position of sternapodeme. The female imago is characterized by coxosternapodeme with a single transparent, well-developed lamella.

##### Description.

**Ault male (*N* = 1).** Total length 1.08 mm. Wing length 0.63 mm. Total length/wing length ratio 1.70.

***Coloration.*** Head and thorax brown, eyes dark brown. Legs pale yellow. Abdominal tergites I-V yellowish, VI-IX yellow-brown.

**Figure 3.**
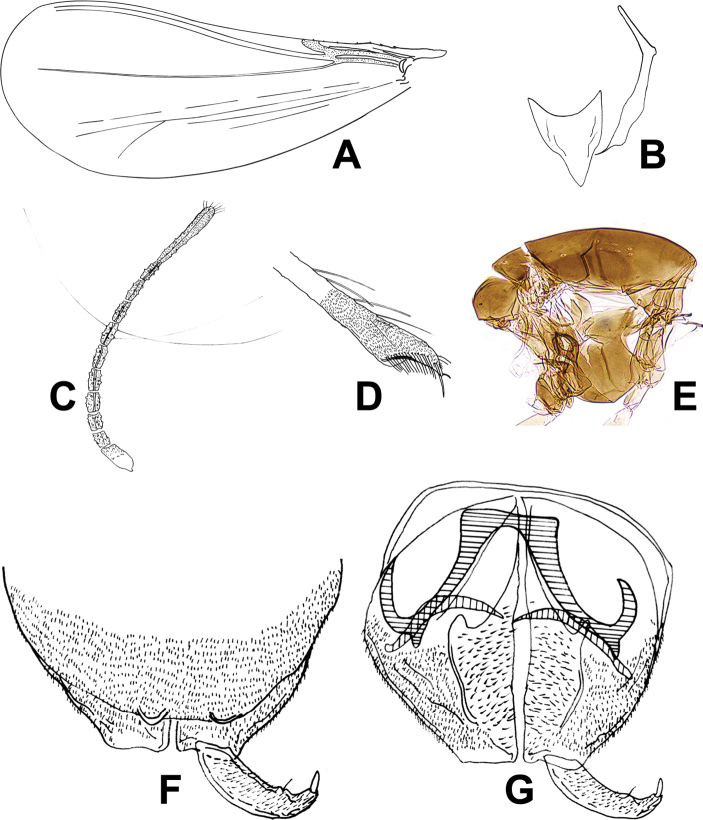
*Corynoneuraincuria* sp. nov., male imago. **A** wing **B** tentorium and cibarial pump **C** antenna **D** legs **E** thorax **F** hypopygium, dorsal view **G** hypopygium, ventral view.

***Head.*** Antenna with eleven flagellomeres, AR 0.31, ultimate flagellomere 96 µm long, slightly expanded apically, with many apical sensilla chaetica (Fig. 3C). Tentorium and cibarial pump as in Figure 3B, tentorium 120 µm long; 12 µm wide. Anterior margin of cibarial pump strongly concave. Palpomeres lost.

***Thorax*** (Fig. 3E). Five dorsocentral setae. Scutellum with two setae.

***Wing*** (Fig. 3A). VR 3.2. Cu/wing length ratio 0.52; Costa 175 µm long, with five setae; Cu 245 µm long; wing width/wing length ratio 0.49.

***Legs.*** Fore trochanter with dorsal keel. Spurs of fore tibia 12 µm long and 7 µm long, spurs of mid tibia 7 and 9 µm long, and spurs of hind tibia 31 µm long and 10 µm long. Width of fore tibia at apex 17 µm, of mid tibia 17 µm, of hind tibia (a) 36 µm. Width of hind tibia ^1^/_3_ from apex (d) 19 µm, elongation length (b) 38 µm, length of maximum thickening (c_1_) 60 µm, total length of thickening (c_2_) 103 µm; hind tibial ratios: a/d 1.89; b/d 2.00; c_1_/d 3.16; c_2_/d 5.42. Hind tibia expanded, with comb of 16 setae, with S-shaped spur (Fig. 3D). Lengths and proportions of legs as in Table 3.

**Table 3.** Lengths (in µm) and proportions of legs segments of male *Corynoneuraincuria* sp. nov. (*N* = 1).

	fe	ti	ta_1_	ta_2_	ta_3_	ta_4_	ta_5_	LR	BV	SV	BR
P_1_	228	255	134	72	41	22	26	0.53	3.83	3.60	1.90
P_2_	323	265	158	73	34	16	28	0.60	4.96	4.35	2.00
P_3_	255	265	134	77	31	17	31	0.51	4.19	3.88	2.00

***Hypopygium*** (Fig. 3F-G). Tergite IX very developed, almost covering the gonocoxite, medially distinctly incurved. Superior volsella with right-angled corner and triangle, and anteromedially separated. Inferior volsella fused with inner margin of gonocoxite bearing many glandular setae. Phallapodeme scalpel-like, apex slightly curved, 31 µm long, and joined with sternapodeme placed caudally. Transverse sternapodeme 14 µm wide, inverted U-shaped with small oral projection, lateral sternapodeme with small attachment point placed and directed caudally. Gonostylus curved, tapering, 23 µm long; megaseta 4 µm long. HR 2.77; HV 4.15.

**Adult female (*N* = 1).** Total length 0.83 mm. Wing length 0.59 mm. Total length/wing length ratio 1.41. Wing length/profemur length ratio 3.61.

***Coloration.*** Head, eyes, and thorax brown. Legs pale yellow. Abdomen yellowish brown.

***Head.*** Tentorium 72 µm long; 7 µm wide. Clypeus with four setae.

**Figure 4.**
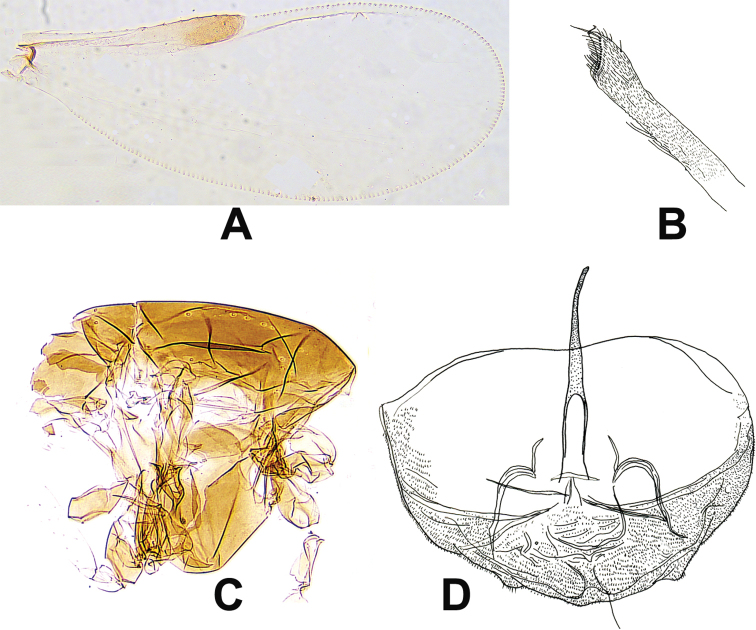
*Corynoneuraincuria* sp. nov., female imago. **A** wing **B** hind tibial apex **C** thorax **D** genitalia.

***Thorax*** (Fig. 4C). Five dorsocentral setae. Scutellum with two setae. Two prealar setae.

***Wing*** (Fig. 4A). Wing broader than in male. VR 2.5. Cu 305 µm long; Cu/wing length ratio 0.52; C 265 µm long; C/wing length ratio 0.45; wing width/wing length ratio 0.49. Costa with 13 setae.

***Legs.*** Fore trochanter with dorsal keel. Spurs of fore tibia 10 µm long, spurs of mid tibia 7 and 12 µm long, and spurs of hind tibia 22 µm and 12 µm long. Width fore tibia at apex of 17 µm, of mid tibia 14 µm, of hind tibia (a) 29 µm. Width of hind tibia ^1^/_3_ from apex (d) 22 µm, elongation length (b) 31 µm, length of maximum thickening (c_1_) 60 µm, total length of thickening (c_2_) 96 µm; hind tibial ratios: a/d 1.32; b/d 1.41; c_1_/d 2.72; c_2_/d 4.36. Hind tibia expanded, with comb of 14 setae, with S-shaped spur (Fig. 4B). Lengths and other proportions of legs as in Table 4.

**Table 4.** Lengths (in µm) and proportions of legs segments of female *Corynoneuraincuria* sp. nov. (*N* = 1).

	fe	ti	ta_1_	ta_2_	ta_3_	ta_4_	ta_5_	LR	BV	SV	BR
P_1_	183	214	106	65	36	17	26	0.50	3.35	3.56	2.50
P_2_	314	230	146	67	31	17	24	0.63	4.96	3.73	1.70
P_3_	199	216	113	70	27	17	24	0.52	3.83	3.67	1.80

***Genitalia*** (Fig. 4D). Tergite IX without long caudal setae. Cercus 26 µm long, 23 µm wide. Notum length 96 µm. Coxosternapodeme with a single transparent well-developed lamella. Seminal capsule 40 µm long, neck 8 µm long, 6 µm wide.

##### Remarks.

This species is closely related to *Corynoneuratokarapequea* Sasa & Suzuki, 1995 by having antenna with eleven flagellomeres, the same shaped sternapodeme and phallapodeme, and a similar gonostylus. The new species can be separated from the latter by having AR 0.31, the inferior volsella almost absent and fused with the inner margin of the gonocoxite, while *C.tokarapequea* has AR 0.62–0.70, the inferior volsella obvious and near rectangular. The new species is also similar to *Corynoneurafloridaensis* Fu & Sæther, 2012 by the antenna with eleven flagellomeres, AR 0.36, same shaped sternapodeme and phallapodeme, but differs from the latter by having a thick transverse sternapodeme, and the gonostylus is strongly curved in *C.floridaensis*.

#### ﻿ Corynoneura longshanensis

Taxon classificationAnimaliaDipteraChironomidae

Fu sp. nov.

BF254C4F-0E38-5236-A856-5CEAFB326DA8

http://zoobank.org/DAFBB15F-09EE-46EA-B6AC-6DBCDE0C3673

Figure 5

##### Type material.

***Holotype*** male (HNU: 17091204HJL), China: Hunan Province, Loudi City, Lianyuan County, Longshan National Forest Park, 27°31'20"N, 111°45'23"E, 674 m a. s. l., 26.Ⅶ.2016, sweep net, leg. Jingli Huang.

##### Etymology.

Named after the type locality.

##### Diagnostic characters.

The male imago is characterized by having an antenna with seven flagellomeres, AR 0.55; superior volsella undeveloped, and inferior volsella with right-angular corner, fused with the inner margin of gonocoxite; sternapodeme inverted U-shaped; phallapodeme scalpel-like, apical curved, in caudal position of sternapodeme.

##### Description.

**Adult male (*N* = 1).** Total length 0.92 mm. Wing length 0.45 mm. Total length/wing length 2.04.

***Coloration.*** Head and thorax brown, legs and abdomen yellowish.

**Figure 5.**
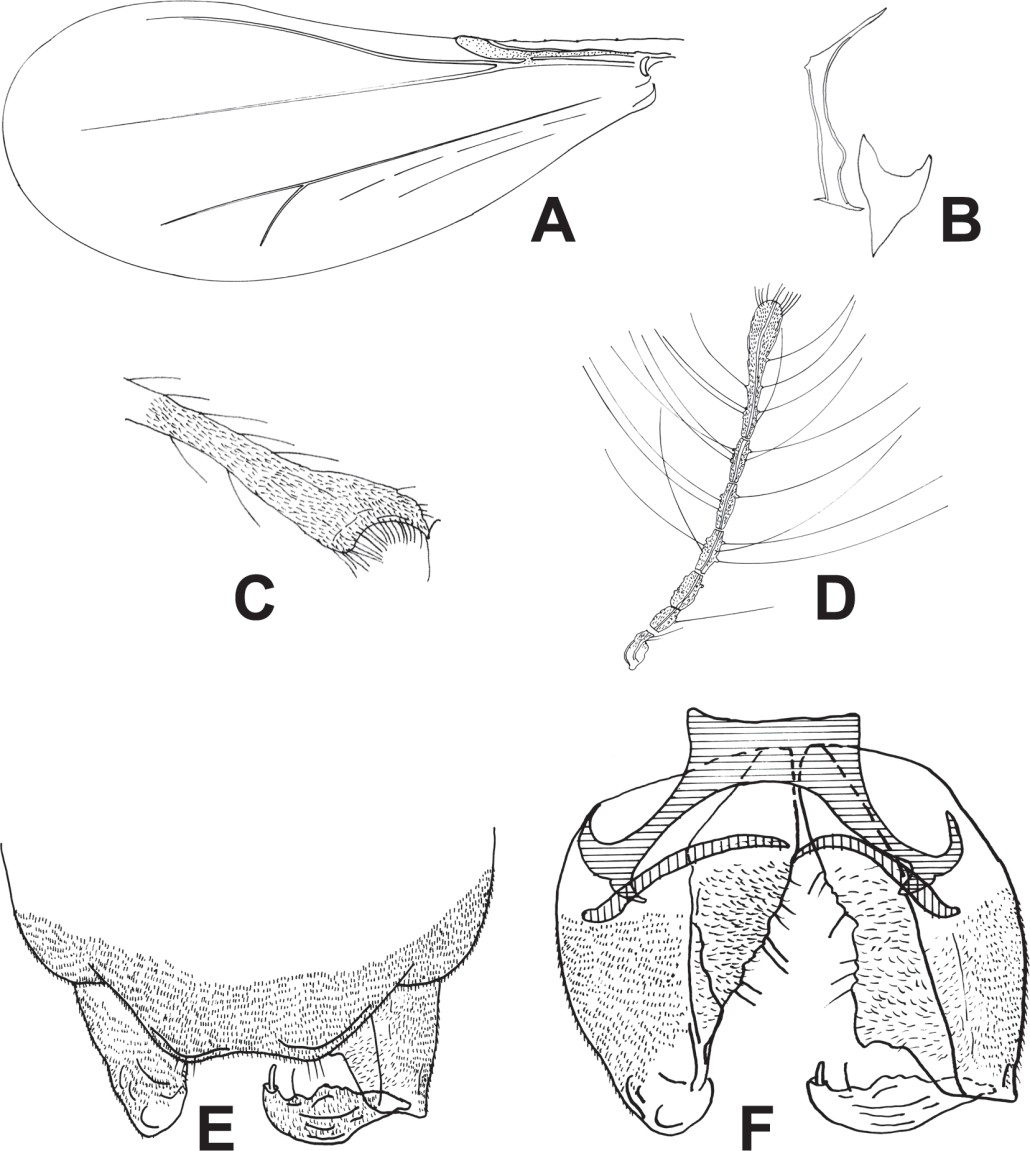
*Corynoneuralongshanensis* sp. nov., male imago. **A** wing **B** tentorium and cibarial pump **C** hind tibial apex **D** antenna **E** hypopygium, dorsal view **F** hypopygium, ventral view.

***Head.*** Antenna with seven flagellomeres, AR 0.55, ultimate flagellomere 89 µm long, ultimate flagellomere distinctly expanded apically, with about 10 apical sensilla chaetica (Fig. 5D). Tentorium and cibarial pump as in Figure 5B, tentorium 96 µm long; 10 µm wide. Anterior margin of cibarial pump strongly concave. Length of palpomeres (in µm): 10; 12; 12; 17; 24. Palpomere 5/3 ratio: 2.0.

***Thorax.*** Five dorsocentral setae. Scutellum with two setae.

***Wing*** (Fig. 5A). VR 3.1. Cu/wing length 0.51; Costa 100 µm long, with five setae; Cu 230 µm long; wing width/wing length ratio 0.47.

***Legs.*** Fore legs lost. Spurs of mid tibia 5 µm and 8 µm long, and spurs of hind tibia 19 µm and 10 µm long. Width of mid tibia at apex 14 µm, of hind tibia (a) 29 µm. Width of hind tibia ^1^/_3_ from apex (d) 14 µm, elongation length (b) 36 µm, length of maximum thickening (c_1_) 60 µm, total length of thickening (c_2_) 79 µm; hind tibial ratios: a/d 2.07; b/d 2.57, 2.10; c_1_/d 4.29; c_2_/d 5.64. Apex of hind tibia obvious expanded, with comb of 16 setae, with S-shaped spur (Fig. 5C). Lengths and proportions of legs as in Table 5.

**Table 5.** Lengths (in µm) and proportions of legs segments of male *Corynoneuralongshanensis* sp. nov. (*N* = 1).

	fe	ti	ta_1_	ta_2_	ta_3_	ta_4_	ta_5_	LR	BV	SV	BR
P_1_	160	180	106	48	26	16	24	0.59	3.91	3.21	1.90
P_2_	209	182	110	48	24	14	22	0.60	4.64	3.55	1.70
P_3_	175	199	91	50	19	12	24	0.46	3.86	4.11	1.80

***Hypopygium*** (Fig. 5E, F). Tergite IX medially slightly incurved. Superior volsella rounded. Inferior volsella with right-angled corner, fused with inner margin of gonocoxite. Phallapodeme 29 µm long, scalpel-like, apex curved, in caudal position of sternapodeme. Transverse sternapodeme 17 µm wide, with small oral projection, inverted U-shape. Gonostylus medially broadened, curved, tapering, 17 µm long; megaseta 5 µm long. HR 2.82; HV 5.40.

##### Remarks.

This new species is similar to *Corynoneurahortonensis* Fu & Sæther, 2012 by having the same shaped sternapodeme and phallapodeme. The new species can be separated from the latter by the broad and thick transverse sternapodeme, and the median part of the gonostylus expanded with a rugged inner edge.

### ﻿Notes on 16S rDNA analysis

**Figure 6.**
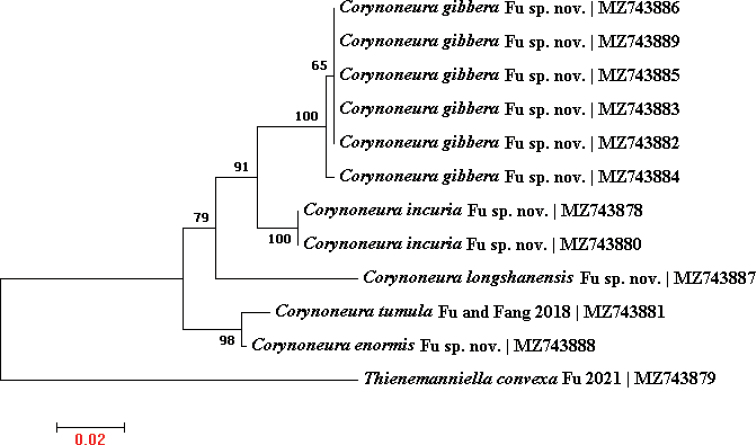
Neighbor-joining Kimura 2 parameter tree based on 16S rDNA of five *Corynoneura* species and *Thienemanniellaconvexa* Fu in Fang et al. (2021). Numbers on branches refer to the percentage of replicate trees in which the associated taxa clustered together in the bootstrap test (500 replicates). Taxa names include scientific names and GenBank accession numbers of corresponding 16S rDNA.

The primary structure of the mitochondrial large ribosomal subunit (16S rDNA) gene is conservative, while the secondary structure shows spiral differences, which are more suitable for systematic studies of species and genera (Simon et al.1994; Harrison 2004). This gene has been successful in identification of chironomids (Cranston et al. 2002; Ekrem et al. 2010). A neighbor-joining tree (Fig. 6) based on 16S rDNA sequences has been proven effective for quickly delimiting and identifying specimens, and supports differentiation of the new species. This study is the first to use 16S rDNA for auxiliary delimitation and identification of specimens in the genus *Corynoneura*.

## ﻿Discussion

The four new species referred in this study share the same morphological features: a transverse sternapodeme inverted U-shape, and the attachment point for the phallapodeme is placed in a caudal position of the sternapodeme. According to Fu et al. (2009), these new species belong to the *celeripes* species group. Additionally, short DNA fragments have been shown to play an important role in the definition of morphospecies (Hebert et al. 2004; Sharkey et al. 2021). In this study, 16S rDNA was used to match male and female individuals from different collections. A similarity of 100% was considered as the same species: thus, the female of *C.incuria* sp. nov. was successfully matched with the male by 16S rDNA.

## Supplementary Material

XML Treatment for Corynoneura

XML Treatment for Corynoneura enormis

XML Treatment for Corynoneura gibbera

XML Treatment for Corynoneura incuria

XML Treatment for Corynoneura longshanensis
